# Construction and Characterization of PDA@MnO_2_-Cored Multifunctional Targeting Nanoparticles Loaded with Survivin siRNA for Breast Tumor Therapy

**DOI:** 10.3390/pharmaceutics18010010

**Published:** 2025-12-21

**Authors:** Jing Zhang, Wenhao Jiang, Lei Hu, Qing Du, Nina Filipczak, Satya Siva Kishan Yalamarty, Xiang Li

**Affiliations:** 1 National Pharmaceutical Engineering Center for Solid Preparation of Chinese Herbal Medicine, State Key Laboratory for the Modernization of Classical and Famous Prescriptions of Chinese Medicine, Innovation and Entrepreneurship College, Jiangxi University of Chinese Medicine, Nanchang 330006, China; jing.zhang@jxutcm.edu.cn (J.Z.); jiangwenhao1@jxutcm.edu.cn (W.J.); hulei@jxutcm.edu.cn (L.H.); 20192019@jxutcm.edu.cn (Q.D.); 2 Center for Pharmaceutical Biotechnology and Nanomedicine, Northeastern University, Boston, MA 02115, USA; nina.filipczak@gmail.com (N.F.); ysskishan@gmail.com (S.S.K.Y.)

**Keywords:** PDA@MnO_2_, survivin siRNA, breast cancer, photothermal therapy

## Abstract

**Objective:** This study aims to engineer a novel nanoparticle formulation for combined tumor therapy, designated as PDA@Mn-siSur-c-NPs, which comprises a polydopamine/manganese dioxide (PDA@MnO_2_) core alongside survivin-targeting siRNA and cyclo(RGD-DPhe-K)-targeting moiety. **Methods**: The PDA@Mn-siSur-c-NPs were constructed and subjected to detailed characterization. Inductively coupled plasma optical emission spectroscopy (ICP-OES) was employed to quantify manganese content. To assess siRNA stability within the system, samples were incubated with 50% fetal bovine serum (FBS) before agarose gel electrophoresis analysis. Additionally, cellular internalization by 4T1 cells and in vitro photothermal conversion efficiency of the formulation were evaluated. ICP-OES was further utilized to investigate the in vivo pharmacokinetics and tissue distribution of manganese. Animal model studies were conducted to assess the anti-breast cancer efficacy of PDA@Mn-siSur-c-NPs in combination with infrared irradiation. **Results**: The newly developed PDA@Mn-siSur-c-NPs demonstrated superior siRNA protection, reduced toxicity, and high photothermal conversion capacity. When combined with photothermal therapy (PTT), these nanoparticles exerted enhanced synergistic anti-tumor effects. Delivery of survivin siRNA resulted in a significant downregulation of survivin protein expression in tumor tissues. Moreover, magnetic resonance imaging (MRI) confirmed that the nanoparticles possess favorable imaging properties. **Conclusions**: This research demonstrates that the integration of PDA@Mn-siSur-c-NPs with PTT holds considerable therapeutic promise for improved breast cancer treatment.

## 1. Introduction

Millions of cancer deaths occur annually worldwide, and new cancer cases are projected to reach 28.4 million by 2040 [1]. Breast cancer has now surpassed lung cancer as the most prevalent cancer globally and is the fifth leading cause of cancer deaths, with an anticipated increase to 4.4 million cases by 2070 [1,2]. Breast cancer accounts for approximately 24.5% of all female cancer cases and 15.5% of cancer deaths [3], making it the primary threat to women’s health globally. For locally invasive cancers, the standard treatment strategy in modern medicine remains surgical resection of the affected area [4]. However, conventional therapies such as surgery, radiotherapy, and chemotherapy are constrained by their poor selectivity, the need for large drug doses, systemic toxicity, and the development of secondary drug resistance resulting from long-term treatment [5].

Photothermal therapy (PTT) is a novel and non-invasive cancer treatment strategy that is highly selective, non-toxic, and easily performed [6]. It uses near-infrared (NIR) lasers, with wavelengths from 700 to 1400 nm, to excite photothermal agents. These agents then convert the absorbed NIR light energy into thermal energy, which causes the ablation and killing of cancer cells. However, PTT has key limitations hampering its broader clinical use. The first near-infrared light only penetrates 2–3 cm, restricting it to superficial tumors rather than deep-seated or metastatic ones [7]; Tumor cells develop thermal resistance via heat shock proteins (e.g., HSP70), reducing therapeutic efficacy [8]; Non-degradable agents lack long-term safety data, and precise temperature control is challenging—excessive heat damages normal tissue, while suboptimal heat fails to eradicate tumors. Additionally, tumor interstitial pressure hinders photothermal agent accumulation [9]. To address the aforementioned limitations and further boost treatment efficacy, synergistic combination therapeutic strategies have increasingly emerged as a research focus in cancer therapy. The synergy between photothermal therapy (PTT) and nanotechnology enables the engineering of multifunctional nanoplatforms capable of co-delivering photothermal effects, therapeutic agents, and even nucleic acid payloads directly to cancer cells in a single system. This innovative integration paves the way for the development of precision-tailored therapeutic regimens that align with the unique biological characteristics of individual patients, advancing toward personalized cancer care [10]. In clinical practice, PTT has been explored for multiple tumor types: for advanced breast cancer, treatment with an 805 nm laser plus indocyanine green and glycated chitosan achieved a 62.5% objective response rate [11], while percutaneous laser ablation for early-stage breast cancer showed an 84% complete tumor ablation rate [7,12]. From clinical advantages, beyond its inherent selectivity and minimal invasiveness, PTT also benefits from the induction of immunogenic cell death for subsequent activation of cytotoxic T lymphocytes (CTLs) to eliminate the tumors and synergize with immunotherapy [9,13]. It also supports real-time MRI/ultrasound guidance for precise targeting [14]. Polydopamine (PDA) is an excellent photothermal material with advantages such as easy functionalization, outstanding biocompatibility, high drug loading capacity, and strong photothermal capacity [15]. PDA-mediated PTT can rapidly increase the local temperature to 54.8 °C within 10 min under NIR irradiation [16], causing the death or damage of tumor cells [17,18]. However, due to tumor heterogeneity, PTT alone is not always ideal for tumor eradication. Research has shown that PTT can increase the thermal stress tolerance of cancer cells, thereby reducing the thermal effect and weakening its efficacy in killing tumor cells [19,20]. The abundant catechol and amine moieties on the PDA layer readily undergo Michael addition or Schiff-base reactions with biomolecules bearing −SH or −NH_2_ groups, enabling efficient conjugation and laying a chemical foundation for PDA-mediated co-delivery of therapeutic agents [21]. Therefore, combining multiple drugs or treatment modalities to create a multimodal, multi-channel synergistic system is crucial for enhancing therapeutic effects and minimizing the toxicity of chemotherapeutic drugs.

The hypoxic state of the tumor microenvironment is a significant factor in tumor growth, invasion, and the development of increased drug resistance [22]. Nanoscale manganese (Mn) has emerged as a promising nanomaterial, drawing considerable attention due to its excellent biocompatibility and potential to mitigate tumor hypoxia and inhibit tumor growth [23,24]. The mechanisms of nanoscale manganese include acting as an oxygen delivery medium to alleviate hypoxia and participating in biochemical reactions to generate reactive oxygen species (ROS), which exert a cytotoxic effect on tumor cells [25,26]. In addition, nanoscale manganese can serve as a contrast agent for magnetic resonance imaging (MRI), reducing the potential toxicity risk to the human body compared to traditional gadolinium (Gd)-based agents. It also enhances imaging contrast and resolution, aiding in the visualization of tumors and their microenvironment [27]. While MRI enables valuable non-invasive visualization, its quantitation limitations for nanomaterials can be complemented by chelate-free radiolabelling strategies for metal oxides, which could be explored in future research for translational refinement [28].

While various strategies have been explored to further optimize the biodistribution of nanotherapeutics—including localized delivery approaches [29], here we prioritized intravenous (IV) injection for our system, considering its high clinical translatability, broad applicability to both primary and metastatic tumors, and compatibility with routine clinical workflows. To further enhance tumor-targeted accumulation within this delivery route, crucially, we have previously reported the functionalization of this nanoscale manganese contrast agent with a cRGD surface modification to achieve targeted delivery to tumor tissue [30].

Survivin, a member of the inhibitor of apoptosis family, is markedly up-regulated in tumor cells following PTT [31]. Such overexpression not only confers rapid thermotolerance but also attenuates apoptosis, ultimately facilitating cancer-cell survival [32]. Thus, the limitations of PTT monotherapy in achieving complete tumor eradication highlight the critical requirement for synergistic combination strategies. RNA interference (RNAi) technology presents both challenges and therapeutic opportunities for breast cancer patients. RNAi can induce the specific degradation of intracellular mRNA, thereby silencing target gene expression [33]. Survivin has been confirmed to be highly expressed in breast cancer cells, making it a potential target for RNAi therapy [33]. The increasing number of siRNA therapies approved by the FDA in recent years attests to the specificity and effectiveness of siRNA-mediated gene silencing [34,35,36]. To surmount the heat tolerance and attenuated cytotoxicity inherent to PTT, we devised a combinatorial strategy that integrates survivin-targeted gene silencing with PTT. By transcriptionally suppressing survivin—the anti-apoptotic protein whose PTT-induced up-regulation underpins thermoresistance—we eliminate adaptive survival signaling, thereby sensitizing tumors to hyperthermia and eliciting a pronounced antitumor response. Nonetheless, siRNA, as a nucleic acid-based drug, faces limitations such as rapid clearance from the circulation and a poor ability to cross biological barriers, which constrain its therapeutic efficacy [37]. We have identified the delivery potential of dendrimers, observing that the addition of a biocompatible segment to the core can improve biodistribution and circulation, enhance surface functionalization, and reduce toxicity and rapid clearance [38]. Recent research indicates that dendrimer surface engineering can also increase drug entrapment, solubility, biodistribution, pharmacokinetics, and stability [39]. Nanocarrier-based delivery systems provide a more effective, stable, and secure means of delivering siRNA. They protect the siRNA from enzymatic degradation and, through specific ligand modifications, ensure accurate targeting and delivery to the desired cells or tissues. Additionally, this approach can reduce non-specific off-target effects, thereby minimizing potential side effects [40].

This research focuses on breast cancer, using a formulation with nanoscale MnO_2_ as the core, coated with polydopamine materials to achieve photothermal therapy and with PAMAM-conjugated lipids to absorb siRNA for silencing survivin protein expression. To further achieve excellent drug-loading performance and precise targeting, the formulation’s surface was modified with cRGD, aiming to achieve a synergistic anti-tumor effect. This multimodal treatment strategy, which combines gene therapy with PTT and MRI, is expected to provide a novel solution for the treatment of breast cancer.

## 2. Materials and Methods

### 2.1. Materials

Dopamine hydrochloride and polyallylamine hydrochloride were obtained from Shanghai Aladdin Bio-Chem Technology Co., Ltd. (Shanghai, China). Potassium permanganate was purchased from Xilong Scientific Co., Ltd. (Shantou, Guangdong, China). L-histidine was sourced from Beijing Solarbio Science & Technology Co., Ltd. (Beijing, China), and L-cysteine from Tokyo Chemical Industry Co., Ltd. (Tokyo, Japan). PAMAM G4 dendrimers were purchased from Sigma-Aldrich (St. Louis, MO, USA). Maleimide-polyethylene glycol 2000-succinimidyl ester (MAL-PEG2000-NHS), methoxypolyethylene glycol 2000-succinimidyl ester (MPEG2000-NHS), and cyclic RGD-polyethylene glycol 3400-succinimidyl ester (cRGD-PEG3400-NHS) were bought from Xi’an Ruixi Biotechnology Co., Ltd. (Xi’an, Shanxi, China). Manganese standard solution was procured from the Tanmo Quality Inspection Standard Material Center (Changzhou, Jiangsu, China). Scramble siRNA (siScr, CAATCGTCGTAACTAGACT) and siRNAs targeting the *survivin* gene (siSur), whose target sequence is CCGTCAGTGAATTCTTGAA, were synthesized by Guangzhou RiboBio Co., Ltd. (Guangzhou, China). All other chemicals were of analytical grade and were used without further purification.

### 2.2. Cells

Mouse 4T1 cells were purchased from Beijing Dingguo Changsheng Biotechnology Co., Ltd. (Beijing, China) The cells were cultured in RPMI 1640 medium with penicillin-streptomycin (pre-supplied, Solarbio, Beijing, China) containing 10% (*v*/*v*) fetal bovine serum (FBS, Yeasen, Shanghai, China) in an incubator at 37 °C with 5% CO_2_.

### 2.3. Animals

Female BALB/c mice (18–20 g) and female Sprague–Dawley rats (200 ± 20 g) were provided by SPF (Beijing) Biotechnology Co., Ltd. (Beijing, China) [SCXK (Jing) 2019-0010]. The animal experiments were conducted accordance to the ethical policies and procedures approved by the Medical Ethics Committee of Jiangxi University of Chinese Medicine (Approval number JZLLSC20250503), which is a general ethical guidance for our team’s overarching anti-tumor drug research program. The animal room was well-ventilated and had a regular 12 h light-dark cycle throughout the experimental period.

### 2.4. Preparation of the PDA@MnO_2_ Cores

MnO_2_ was prepared by the revised reduction method [41]. First, the solution of 0.02 M potassium permanganate was added dropwise to 0.4 M polyallylamine hydrochloride solution at a ratio of 2.2:1 under stirring at 800 rpm in a 30 °C water bath. After 30 min, the resulting MnO_2_ was collected and dialyzed against deionized water for 3 h. Separately, dopamine hydrochloride, L-histidine, and L-cysteine were each prepared as we previously reported [21]. Briefly, MnO_2_ solution was dropped into a reaction system containing ammonia water and 30% ethanol (*v*/*v* 1:3) at 25 °C. Then dopamine hydrochloride and histidine (molar ratio = 1.1:1) were added to the reaction system. After 1 h of reaction, the same concentration of L-cysteine solution was added. The reaction proceeded for an additional 5 h, after which the mixture was dialyzed in 0.1 M carbonate buffer for 3 h to yield hybrid PDA-coated MnO_2_ cores (PDA@MnO_2_). The product was stored at 4 °C in the dark for further use. Mn content, particle size, and zeta potential were determined using an inductively coupled plasma optical emission spectrometer (ICP-OES, 700ES, Varian, Inc., Schiller Park, IL, USA) and a Malvern ZEN3690 particle size analyzer (Malvern Panalytical Ltd., Malvern, Worcestershire, UK), respectively.

### 2.5. Preparation of PDA@Mn-siSur-c-NPs

The absolute ethanol solution of MAL-PEG2000-NHS at 34.5 mM was dropped into 0.1 M sodium bicarbonate (NaHCO_3_) solution containing PAMAM at 5.0 mM. The mixture was stirred at 800 rpm at room temperature for 3 h, facilitating the reaction between NHS and amino groups (−NH_2_) to proceed, thus yielding MAL-PEG2000-PAMAM. MAL-PEG2000-PAMAM solution was slowly added to the PDA@MnO_2_ solution and allowed to react at room temperature for 12 h, to facilitate the reaction between maleimide (MAL) and thiol groups (−SH) in the hybrid PDA@MnO_2_ cores. The reaction mixture was then collected and dialyzed against deionized water, yielding PAMAM-PEG2000-coated cores (PAM/PDA@Mn).

MPEG2000-NHS and cRGD-PEG3400-NHS (molar ratio = 10:1) in anhydrous ethanol were added dropwise to PAM/PDA@Mn, and the mixture was stirred at 800 rpm at room temperature for 12 h to yield cRGD modified PAM/PDA@Mn (c-PAM/PDA@Mn). Subsequently, survivin siRNA (siSur) was mixed with c-PAM/PDA@Mn at different N:P ratios (the ratio between the amine groups of the ionizable lipid (N) and the phosphate groups of the cargo (P)) and incubated at 37 °C on a shaker for 30 min to form PDA@Mn-siSur-c-NPs. As a negative control for in vivo investigation, siSur was replaced with scrambled siRNA (siScr), generating PDA@Mn-siScr-c-NPs. PDA@Mn-siSur-NPs were prepared as the same protocol as PDA@Mn-siSur-c-NPs, without adding cRGD-PEG3400-NHS to the coating lipids.

### 2.6. Characterization of PDA@Mn-siSur-c-NPs

#### 2.6.1. Gel Retardation Assay

The encapsulation efficiency of siRNA within c-PAM/PDA@Mn was evaluated via agarose gel electrophoresis. The nanoparticle suspension was added to the preprepared siRNA solution at different N:P ratios (8:1, 6:1, 5:1, 4:1, 3:1, 2:1, 1:1), and 20 μL of samples were analyzed by gel electrophoresis using E-Gel™ General Purpose 2% Agarose Gels containing ethidium bromide (EB) (Invitrogen, Waltham, MA, USA) at 60 V for 15 min with E-Gel™ Power Snap Electrophoresis Device (Invitrogen, MA, USA). Bands of siRNA were visualized under UV light with E-Gel™ Power Snap Camera (Invitrogen, MA, USA).

#### 2.6.2. Size, Zeta Potential, and Morphology

The particle size and zeta potential of MnO_2_, PDA@Mn, and PDA@Mn-siSur-c-NPs were determined using a Malvern Nanoparticle Size Analyzer (ZEN3690, Malvern Panalytical Ltd, Malvern, Worcestershire, UK). For transmission electron microscopic (TEM) (JEM-2100, JEOL Ltd., Tokyo, Japan) observation, MnO_2_, PDA@Mn, and PDA@Mn-siSur-c-NPs were diluted with ultra-pure water, dropped onto copper grids containing a carbon film (400 mesh), and allowed to air-dry. Morphological analysis and imaging were conducted using a Zeiss Merlin scanning electron microscope (Carl Zeiss AG, Oberkochen, Germany) operated at 10 kV, with a spatial resolution of approximately 1.3 nm. A Bruker Flash 6ǀ60 Energy-Dispersive Spectrometer (Bruker Corporation, Billerica, MA, USA) was employed to assay formulation composition.

#### 2.6.3. Determination of Manganese Content

The manganese content of the samples was quantified using ICP-OES. Instrument parameters were set as follows: radio frequency power, 1.5 kW; high-purity argon gas flow rate, 1.5 L/min; nebulizer temperature, 2.5 °C; and auxiliary gas flow rate, 0.8 L/min. The peristaltic pump speed was adjusted to 15 rpm, resulting in a sample introduction rate of 1 mL/min. Measurements were performed in triplicate.

#### 2.6.4. Simulated Serum Stability

Equal volumes of PDA@Mn-siSur-c-NPs solution and FBS were mixed and incubated at 37 °C in a dark water bath for 30 min with shaking. At predetermined time points (0, 1, 2, 4, 8, 12, and 24 h), 10 µL aliquots were withdrawn and mixed with heparin solution (heparin sodium to siSur ratio = 26 IU/µg) [42] to release the siRNA. The mixtures were incubated at 37 °C for 30 min with shaking. The degree of siRNA degradation was analyzed by agarose gel electrophoresis, with free siRNA used as a control. The gels were observed and photographed using a gel imaging system.

#### 2.6.5. Cellular Uptake Evaluation

To evaluate the cellular uptake of PDA@Mn-siSur-NPs and PDA@Mn-siSur-c-NPs, 4T1 cells were seeded in 6-well plates at a density of 2 × 10^6^ cells/well and incubated overnight at 37 °C in a humidified atmosphere containing 5% CO_2_. The cells were then exposed to nanoparticles (Mn concentration = 7.2 µg/mL) for either 5 or 15 min. Following incubation, the cells were washed three times with PBS, collected, and transferred into conical flasks containing 5 mL of concentrated nitric acid for digestion. The intracellular manganese content was determined by ICP-OES using the same parameters described above.

#### 2.6.6. In Vitro Photothermal Conversion Effect

Ultrapure water served as the control, while MnO_2_, PDA@Mn and PDA@Mn-siSur-c-NPs suspensions (28 μg/mL of Mn) were tested—including 2-fold, 4-fold, and 8-fold diluted samples of PDA@Mn stock solution. All the samples were irradiated with an 808 nm laser at a power density of 2.5 W/cm^3^. The temperature of each solution was recorded at 30 s intervals using a TES-1310 thermocouple thermometer (TES-1310, Suzhou Runqi Electronic Technology, Suzhou, Jiangsu, China) immersed in the solution.

PDA@Mn and PDA@Mn-siSur-c-NPs stock solution was subjected to irradiation with a 2.5 W/cm^3^ infrared laser (LWIRPD-15F, Beijing Laserwill Optoelectronic Technology Co., Ltd., Beijing, China) in a cyclic manner, which consisted of 10 min of irradiation (heating) followed by 10 min of pause (cooling). This heating-cooling cycle was repeated a total of 6 times. The temperature changes were continuously recorded to generate a time-temperature curve.

#### 2.6.7. Pharmacokinetic Dectectioin

Female Sprague–Dawley rats were randomly divided into a PDA@Mn-siSur-NPs group and a PDA@Mn-siSur-c-NPs group (*n* = 3 per group). The rats were fasted for 12 h before the experiment. Each group received a tail vein injection of nanoparticles at a dose of 0.27 mg/kg (manganese equivalent). Blood samples were collected from the orbital vein at 0.017, 0.083, 0.25, 0.5, 1, 2, 4, 8, 12, and 24 h post-injection. The samples were digested, and the manganese content was quantified. The pharmacokinetic parameters for each formulation were calculated using PKSolver 2.0 software.

#### 2.6.8. Tissue Distribution Assay

Tumor-bearing female mice were divided into PDA@Mn-siSur-NPs and PDA@Mn-siSur-c-NPs groups (*n* = 3 per time point) and administered nanoparticles via tail vein injection at a dose of 0.675 mg/kg (manganese equivalent). Samples were collected from each group at 0.5, 2, and 8 h post-injection. At each time point, mice were euthanized, and the heart, liver, spleen, lungs, kidneys, and tumor tissues were excised, rinsed, dried, and weighed. Tissue samples were then cut, digested, and assessed for manganese content using ICP-OES.

#### 2.6.9. Tumor Tissue Temperature Measurement

Tumor-bearing female mice were divided into three groups: model (saline injection), high-dose PDA@Mn-siSur-c-NPs (0.945 mg/kg Mn equivalent), and low-dose PDA@Mn-siSur-c-NPs (0.405 mg/kg Mn equivalent). To enhance nanoparticle enrichment in tumor tissues and maximize the detectable difference in photothermal response between groups, mice in the treated groups received three consecutive intravenous injections at the respective doses. Immediately after the third injection to avoid signal attenuation, tumor sites were irradiated with an infrared laser (2.3 W/cm^3^) for 80 s. Tumor temperatures were recorded using a Testo 865 thermal imager (Testo Instruments International Trading (Shanghai) Co., Ltd., Shanghai, China) and compared across groups with different concentrations of Mn.

#### 2.6.10. MRI Contrast Test Assay

For MRI detection, a 0.35 T SUPER NOVA P3 scanner was used, equipped with mouse body coil. Mice were placed in a centrifuge tube inverted on cardboard for T_1_-weighted signal acquisition. The T_1_-weighted sequence parameters were set as follows: TR = 500 ms, TE = 10 ms, flip angle = 90°, field of view (FOV) = 30 × 30 mm, matrix = 256 × 256, slice thickness = 1 mm. Prior to imaging, mice were anesthetized with 1–2% isoflurane in oxygen mixture (flow rate: 1 L/min) administered via a nose cone; meanwhile, the mice’s body temperature was maintained at 36.5–37.5 °C using a heating pad integrated into the scanner bed. For in vivo imaging, PDA@Mn-siSur-c-NPs (0.405 mg/kg Mn equivalent) was injected intravenously into mice, and MR spectra were acquired at multiple time points post-injection.

### 2.7. Anti-Tumor Evaluation

4T1 cells (1 × 10^6^ cells/mouse) were inoculated into the mammary fat pads of female BALB/c mice. When tumor volumes reached ~100 mm^3^, thirty-five mice were randomized into seven groups according to random number table: PDA@Mn-siScr-c-NPs, PDA@Mn-siScr-c-NPs+NIR, PDA@Mn-siSur-c-NPs, PDA@Mn-siSur-c-NPs+NIR, Free siSur, Free siSur+NIR, and Model groups. All nanoparticle treatments were administered via the tail vein at 0.45 mg/kg (Mn equivalent) once every 7 days for a total of three doses. Irradiation groups received 5 min laser irradiation (2.3 W/cm^3^) at the tumor site 2 h after each injection. Tumor volumes and body weights were recorded throughout the treatment period. Tumor volume (*V*) was calculated using the following formula:

$$V=\frac{W^{2}\times L}{2}$$

where *V* is the tumor volume, *L* is the longest diameter of the tumor, and *W* is the shortest diameter of the tumor.

Ten days after the final injection, mice were euthanized, and tumor, heart, liver, spleen, lung, and kidney tissues were collected for subsequent analyses. Blood samples were obtained from the orbital plexus into heparinized tubes and centrifuged at 5000 rpm for 5 min to separate plasma. Tumors were excised and weighed. Portions of the tumor were fixed in 4% formalin, embedded in paraffin, and subjected to hematoxylin and eosin (H&E) staining following standard protocols. The remaining tumor samples were snap-frozen in liquid nitrogen and stored at −80 °C for Western blot assays.

### 2.8. Western Blot

Tumor tissues from each group were homogenized in lysis buffer, and total protein content was determined using a BCA assay kit (Jiangsu Cowin Biotech Co., Ltd., Taizhou, Jiangsu, China). Equal amounts of protein from each group were separated by SDS-PAGE and transferred to nitrocellulose membranes. After blocking, membranes were incubated overnight at 4 °C with rabbit anti-mouse survivin primary antibody. Following washing, the membranes were incubated for 1 h at room temperature on a shaker with goat anti-rabbit secondary antibody. Detection was performed using a chemiluminescent substrate, with a 3 min reaction time before darkroom exposure. β-Actin was used as the internal control.

### 2.9. Measurement of Biochemical Parameters

A fully automated biochemical analyzer (BS-1000M, Mindray, Shenzhen, Guangdong, China) was used to quantify serum levels of alanine aminotransferase (ALT), aspartate aminotransferase (AST), blood urea nitrogen (BUN), and creatinine (CRE) from collected blood samples.

### 2.10. H&E Staining

After the anti-tumor efficacy experiment, heart, liver, spleen, lung, kidney, and tumor tissues were collected, fixed in polyformaldehyde for 24 h, then rinsed for 12 h. They were then embedded and sectioned. The slides were then stained using an H&E kit (Solarbio, Beijing, China) and observed under the Eclipse NI-E/Ni-U microscope (Nikon, Tokyo, Japan).

### 2.11. Statistical Analysis

All quantitative measurements were conducted with at least three replicates and are presented as means ± standard deviations (SD). All data were statistically analyzed by GraphPad Prism 10.6.0: two-group comparisons via Mann–Whitney U test (two-tailed), and multi-group comparisons via Kruskal–Wallis H test (two-tailed) with Dunn’s post hoc test. *p* < 0.05 was considered statistically significant.

## 3. Results and Discussion

### 3.1. Preparation and Characterization of PDA@Mn-siSur-c-NPs

**Figure 1:**
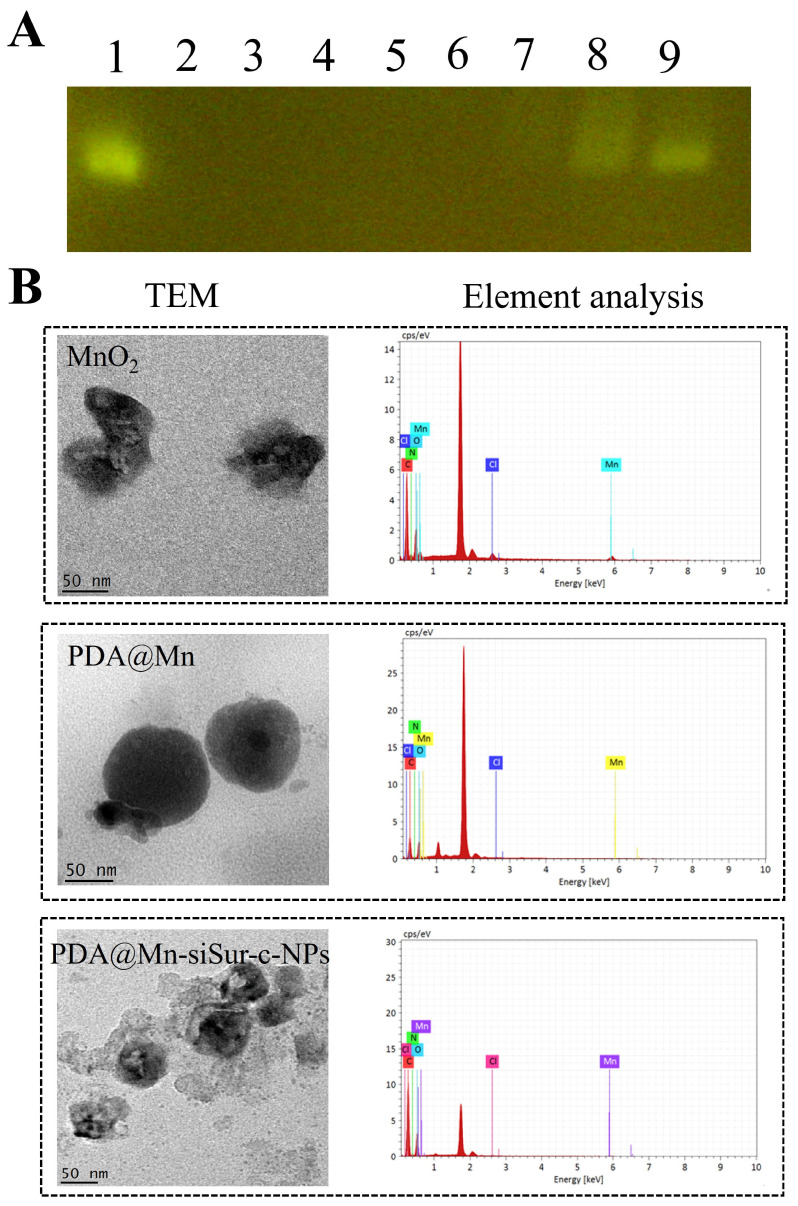
(**A**) Agarose gel electrophoresis of c-PAM/PDA@Mn to siRNA (Lane 1: Free siRNA; Lane 2: c-PAM/PDA@Mn; Lane 3–9: PDA@Mn-siSur-c-NPs N:P 8:1, 6:1, 5:1, 4:1, 3:1, 2:1; 1:1); (**B**) Transmission electron microscopic images of MnO_2_, PDA@Mn, and PDA@Mn-siSur-c-NPs along with their surface elements.

Agarose gel electrophoresis demonstrated that, using free siRNA as a control, the N:P ratio was correlated with the encapsulation efficiency of siRNA in c-PAM/PDA@Mn. It was found that complete encapsulation of siRNA was achieved when the N:P ratio was ≥4, leading to the selection of 4:1 as the optimal N:P ratio (Figure 1A). TEM images (Figure 1B) revealed that all formulations exhibited a spherical or quasi-spherical morphology and were uniformly dispersed. The hydrodynamic diameters, polydispersity index (PDI), and zeta potential values for MnO_2_, PDA@Mn, and PDA@Mn-siSur-c-NPs are shown in Table 1 and Figure S1.

The manganese content in PDA@Mn-siSur-c-NPs, as measured by ICP-OES, was found to be 27.89 ± 3.89 μg/mL.

Surface elemental analysis showed that the normalized mass percentages of Mn, C, O, N, and Cl in MnO_2_ were 22.82%, 46.49%, 20.23%, 7.72%, and 2.74%, respectively (Figure 1B). In contrast, the PDA@Mn-siSur-c-NPs preparation, obtained after coating MnO_2_ with PDA and PEG–PAMAM, contained 1.33%, 56.14%, 34.41%, 7.76%, and 0.36% Mn, C, O, N, and Cl, respectively. Notably, the mass proportions of Mn and Cl were markedly reduced following surface modification. X-ray photoelectron spectroscopy was also performed for MnO_2_ and PDA@Mn, which confirmed the entrapped Mn chemical state is +4 (characteristic of MnO_2_, Figure S2). These results indicated that PDA and PEG–PAMAM effectively coated the MnO_2_ surface, thereby masking the Mn signal from MnO_2_ and polyallylamine hydrochloride, as well as the Cl signal from dopamine hydrochloride.

### 3.2. Stability in Simulated Serum

**Figure 2:**
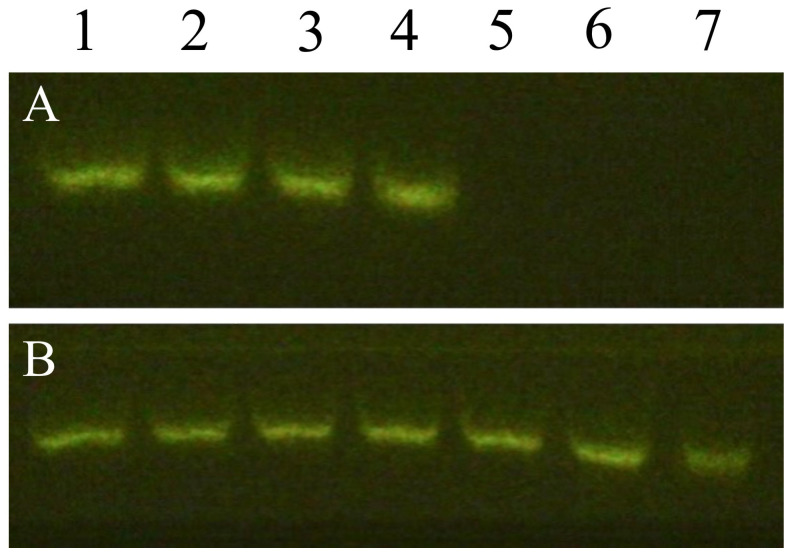
Gel electrophoresis diagrams of Free siSur (**A**) and PDA@Mn-siSur-c-NPs (**B**) (1–7: 0, 1, 2, 4, 8, 12, and 24 h, respectively).

Free siSur was found to have poor stability and was susceptible to degradation and was completely degraded within 8 h in a 50% FBS solution, with no bright bands observed following gel electrophoresis (Figure 2A). In contrast, PDA@Mn-siSur-c-NPs showed a relatively distinct siSur band even after 24 h in 50% FBS (Figure 2B), likely due to the protective effect of its outer layer, which comprised polydopamine, long-circulating polyethylene glycol, and the PAMAM layer [43,44,45].

### 3.3. Cellular Uptake

For the cellular manganese uptake assay, equal amounts of manganese-containing PDA@Mn-siSur-NPs and PDA@Mn-siSur-c-NPs were added to 4T1 cells, followed by incubation at 37 °C with 5% CO_2_ for 5 and 15 min, respectively (Table 2). The results showed that compared to PDA@Mn-siSur-NPs, PDA@Mn-siSur-c-NPs were more readily taken up by cells in these short incubation periods. Specifically, manganese uptake from PDA@Mn-siSur-c-NPs was 1.19- and 1.26-fold higher than that from PDA@Mn-siSur-NPs at the 5- and 15 min sampling time points, respectively.

### 3.4. In Vitro Photothermal Conversion Capability

**Figure 3:**
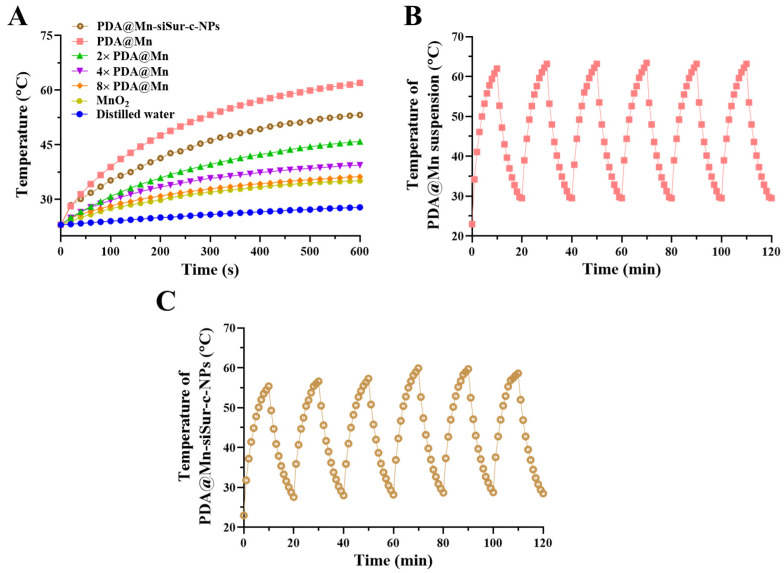
(**A**) Temperature-time curve for each preparation. (**B**) The change in temperature for PDA@Mn suspension after repeated heating cycles. (**C**) The change in temperature for PDA@Mn-siSur-c-NPs after repeated heating cycles.

In photothermal therapy, the heat conversion effect of photothermal materials is used to raise the local tissue/cell temperature above 40 °C, leading to a series of reactions, such as protein denaturation, in tumor cells, resulting in their death. Polydopamine is an ideal photothermal material. A control group containing ultrapure water and experimental groups with varying concentrations of PDA@Mn were prepared. For each formulation, 1 mL was placed in quartz dishes and continuously irradiated with an 808 nm laser at a power density of 2.5 W/cm^3^. Temperature changes were recorded in real time, and time-temperature curves were generated. As shown in Figure 3A, after 10 min of laser irradiation, the temperatures of the ultrapure water control, PDA@Mn, 2-fold diluted PDA@Mn, 4-fold diluted PDA@Mn, and 8-fold diluted PDA@Mn reached 27.8, 62.0, 45.9, 39.4, and 36.2 °C, respectively. A concentration-dependent increase in photothermal heating was observed, with higher PDA@Mn concentrations producing greater temperature elevation (ΔT).

By subjecting the original PDA@Mn Solution group to repeated irradiation with a 2.5 W/cm^3^ infrared laser for 10 min, followed by a 10 min cooling period without irradiation in a cyclic manner, the formulation was able to maintain the ability to repeatedly increase in temperature under these conditions, demonstrating a stable photothermal conversion capability (Figure 3B).

After entrapped by lipid layers to form PDA@Mn-siSur-c-NPs, the photothermal conversion ability remains basically stable (Figure 3A), as the NIR absorption structure of the PDA core is not destroyed, siSur and targeting legand do not interfere with 808 nm light absorption. Although surface functionalization marginally reduces ΔT compared to PDA@Mn, the thin coating layer does not significantly compromise the core’s light absorption, which is sufficient to induce irreversible damage to cancer cells (via protein denaturation and membrane disruption) while minimizing damage to normal tissues (which can tolerate temperatures below 42 °C. The cyclic photothermal stability of PDA@Mn-siSur-c-NPs is also significantly maintained after 6 heating-cooling cycles (Figure 3C).

This study confirmed that under NIR light irradiation, the local temperature can be rapidly increased to 53.2 °C within 10 min, with the rate of temperature rise and the final temperature being directly proportional to the concentration. The overall performance does not compromise the application requirements for synergistic photothermal-gene therapy.

### 3.5. Pharmacokinetics

**Figure 4:**
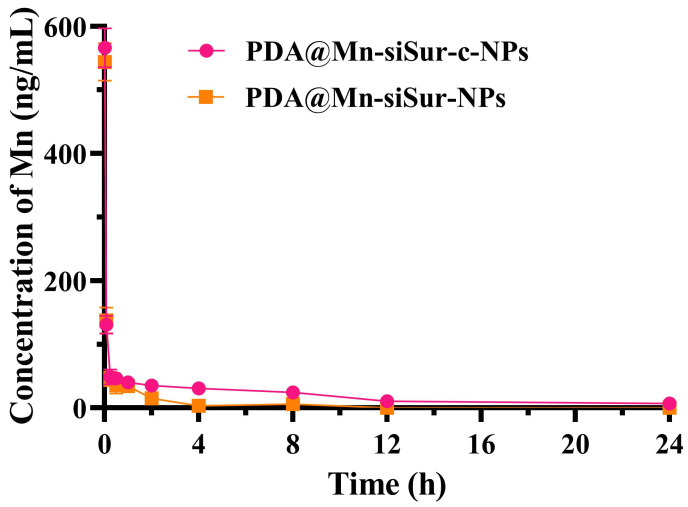
Blood drug concentration-time curve for each preparation (*n* = 3).

The concentration of Mn in blood, either alone or incorporated within the nanoparticles, was determined at different time points by ICP-OES. As shown in Figure 4, PDA@Mn-siSur-NPs were eliminated substantially faster than cRGD-modified PDA@Mn-siSur-c-NPs. Analysis of key pharmacokinetic parameters (Table 3) showed that the area under the concentration-time curve (AUC_0→t_) and the mean residence time (MRT) of the PDA@Mn-siSur-c-NPs were increased by 2.8- and 3.0-fold, respectively. At the same time, the drug clearance rate (CL) and the apparent volume of distribution (V_d_) were decreased by 68.7% and 18.9%, respectively, compared to those of the PDA@Mn-siSur-NPs. These findings suggested that the PDA@Mn-siSur-c-NPs exhibit superior long-circulating capabilities in vivo relative to PDA@Mn-siSur-NPs.

### 3.6. Tissue Distribution

**Figure 5:**
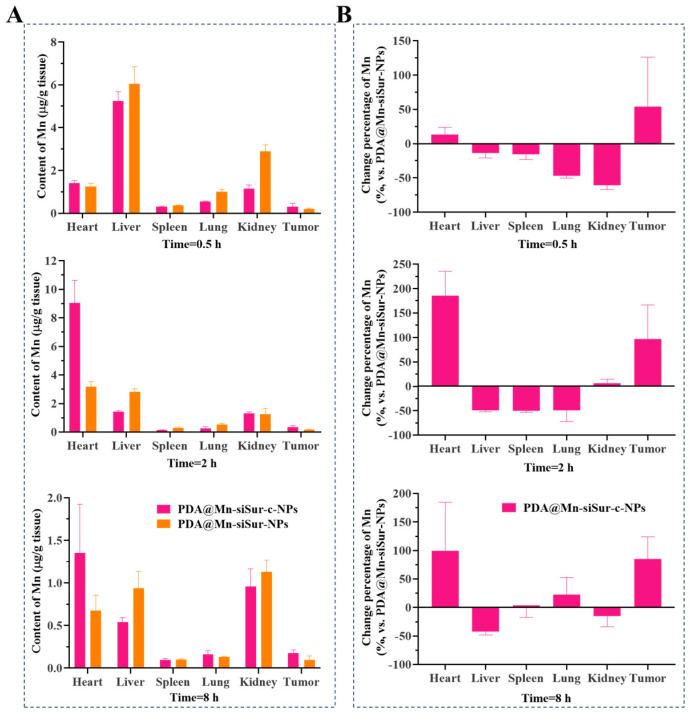
(**A**) The tissue distribution of Mn in PDA@Mn-siSur-NPs and PDA@Mn-siSur-c-NPs at different time points; (**B**) Change (%) of Mn tissue distribution vs. PDA@Mn-siSur-NPs at different time points (*n* = 3).

Normalized tissue distribution data were obtained for each organ, using PDA@Mn-siSur-NPs as the reference formulation, and the relative change rate in PDA@Mn-siSur-c-NPs contents was calculated. As shown in Figure 5, the concentration of PDA@Mn-siSur-c-NPs in tumor tissue increased continuously within the first 4 h and remained significantly higher than that of PDA@Mn-siSur-NPs at 8 h. These results indicated that PDA@Mn-siSur-c-NPs possess enhanced tumor-targeting capability.

Surface coating is a widely used method for improving the relaxation rate of manganese oxide nanoparticles, with approaches including polymer functionalization, silica coating, and phospholipid modification [46,47]. The results of the surface elemental analysis confirmed the success of the coating process. Grafting cRGD, which has no cytotoxicity, onto metal nanoparticles can endow them with targeting capabilities. One of the characteristics of metal nanoparticles is their large surface area, which facilitates ligand modification [48]. In this study, the surface of manganese nanoparticles was functionalized with cRGD, which not only provided tumor-targeting properties but also preserved MRI contrast performance. Compared to unmodified PDA@Mn, PDA@Mn-siSur-c-NPs demonstrated longer circulation time in vivo, which is primarily ascribed to the PEG-mediated regulation of the protein corona. The surface PEGylation of PDA@Mn-siSur-c-NPs could induces the formation of a dense “brush conformation” of PEG chains [49], which selectively enriches dysopsonins while minimizing the adsorption of opsonins. Then the PEG chains not only enhance the colloidal stability of the nanoparticles in blood, but effectively blocks recognition by the mononuclear phagocyte system (MPS) and preserves the structural integrity of the “stealth” protein corona during circulation [50]. In contrast, unmodified PDA@Mn readily forms an opsonin-rich, clearance-promoting protein corona that triggers rapid MPS-mediated elimination. The PEG-induced protein corona composition also ensured the sustained and effective silencing of survivin protein expression. siRNA-mediated silencing of survivin protein expression within tumors effectively inhibited tumor growth, with delivery efficiency and better tissue distribution remaining a critical factor for therapeutic success [51].

### 3.7. Tumor Tissue Thermometry

**Figure 6:**
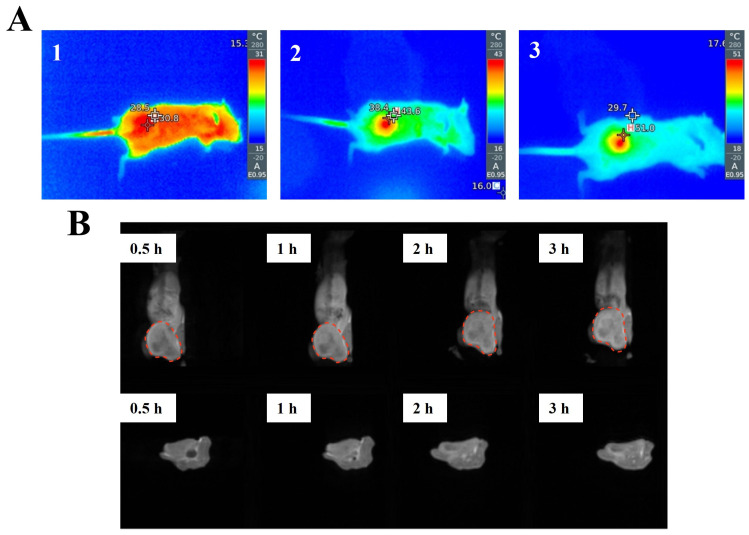
(**A**) Thermography of mice from the model group (1), low-dose PDA@Mn-siSur-c-NPs-treatment group (2), and high-dose PDA@Mn-siSur-c-NPs-treatment group (3) (Mn equivalents of 0.405 and 0.945 mg/kg, respectively). (**B**) Mouse MRI after the intravenous injection of PDA@Mn-siSur-c-NPs at different time points (The region of interest encircled corresponds to the tumor site).

As shown in Figure 6A, immediately after administration and exposure to light, the tumor tissue temperature of the mice in the model group was 30.8 °C. For the low-dose and high-dose administration groups, the tumor tissue temperature increased to 43.6 and 51 °C within 80 s, respectively, values that were 1.42- and 1.66-fold higher than those of the model group.

### 3.8. MRI Contrast Enhancement Profiles

Next, we investigated the ability of the formulation to enrich in tumor tissue and its imaging capability over different periods by administering PDA@Mn-siSur-c-NPs to tumor-bearing mice via tail vein injection (Figure 6B). MRI observations from the local tumor coronal and sagittal planes indicated that PDA@Mn-siSur-c-NPs increasingly accumulated over time at the tumor site and demonstrated a more pronounced imaging effect in the presence of manganese.

The core manganese dioxide not only serves as an oxygen transfer medium to improve hypoxic conditions but also exhibits high-quality MRI effects. Mn(II) ions may be a key factor in the strong MRI capability of manganese oxide nanoparticles, as the five unpaired electrons in their 3d orbitals can produce a large magnetic moment and cause nearby water proton relaxation, which means that manganese oxide nanoparticles are potential candidates for T_1_-weighted MRI contrast agents [52].

### 3.9. Anti-Tumor Efficacy

**Figure 7:**
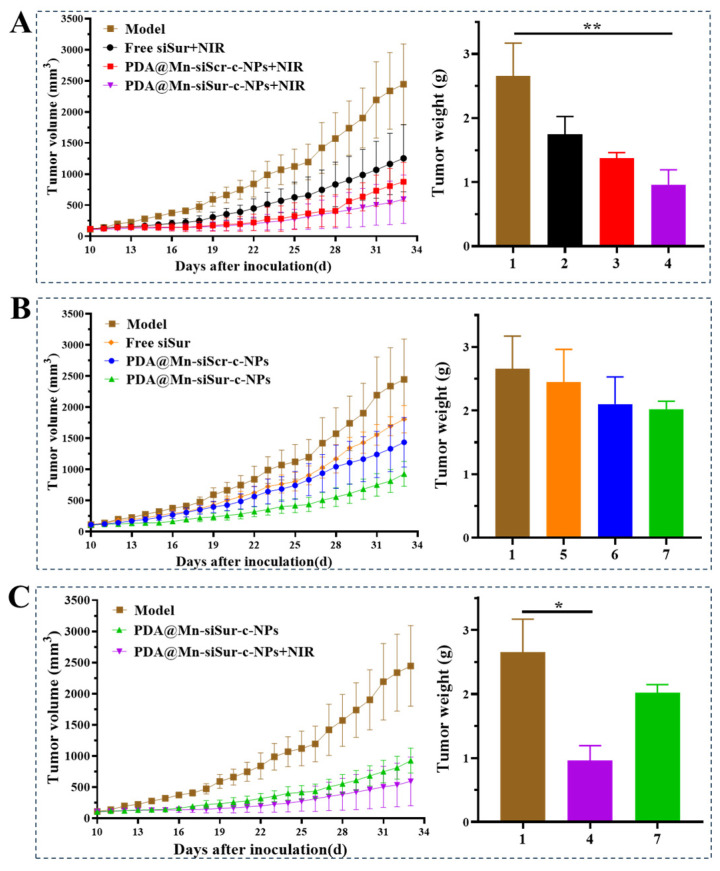
Compared with model group, tumor growth curve of groups treated with different preparations combined with NIR (**A**); groups treated with different preparations without combination of NIR (**B**); groups treated with PDA@Mn-siSur-c-NPs with and without NIR (**C**), with the tumor weight measured on the last day among different comparison was shown (1: Model group; 2: Free siSur+NIR; 3: PDA@Mn-siScr-c-NPs+NIR; 4: PDA@Mn-siSur-c-NPs+NIR; 5: Free siSur; 6: PDA@Mn-siScr-c-NPs; 7: PDA@Mn-siSur-c-NPs) (*n* = 5, * *p* < 0.05, ** *p* < 0.01).

The group treated with PDA@Mn-siSur-c-NPs under NIR radiation (PDA@Mn-siSur-c-NPs+NIR) exhibited the best tumor growth suppression effect (Figure 7A), with a tumor inhibition rate of 63.8%. The inhibitory effect was 1.34 and 1.89 times that of the PDA@Mn-siScr-c-NPs+NIR and Free siSur+NIR groups, respectively. The PDA@Mn-siSur-c-NPs+NIR formulation group exhibited the best tumor inhibition efficiency than the PDA@Mn-siScr-c-NPs+NIR group, likely attributable to the presence of siSur, when compared with the model group (** *p* < 0.01).

A comparison of the non-irradiated groups (Figure 7B) revealed that the tumor inhibition rate of the PDA@Mn-siSur-c-NPs was 23.71%, which did not significantly differ from that of the Free siSur and PDA@Mn-siScr-c-NPs, indicating that photothermal inversion is important for achieving a good tumor inhibitory effect.

A comparative analysis between the irradiated and non-irradiated groups (Figure 7C) revealed that the tumor inhibition rate of PDA@Mn-siSur-c-NPs+NIR was 2.68 times that of PDA@Mn-siSur-c-NPs, indicating that the tumor inhibition rate in the irradiated group of mice was superior to that in the corresponding non-irradiated treatment group.

This prominent synergistic antitumor effect is closely related to the multi-functional integrated design of our nanosystem, which differs significantly from the general landscape of most existing photothermal nanocarriers—unlike conventional photothermal nanoparticles that typically focus solely on PDA- or metal-based photothermal conversion with limited additional therapeutic or diagnostic functions [53,54], our PDA@Mn-siSur-c-NPs innovatively integrate PDA-mediated PTT, survivin siRNA-based gene therapy, and MnO_2_-derived multi-modal bioactivities, enabling multi-pronged tumor inhibition rather than relying solely on heat-induced cell necrosis.

Additionally, for the existing photothermal nanoparticles lacking diagnostic capabilities, making real-time monitoring of tumor localization and nanoparticle accumulation difficult during treatment [55,56,57], while our system designed MnO_2_ component, serving as an MRI contrast agent for non-invasive tumor visualization.

Collectively, the superior efficacy of PDA@Mn-siSur-c-NPs+NIR stems from its innovative design providing a comprehensive and clinically relevant strategy for breast cancer treatment.

### 3.10. Body Weight

**Figure 8:**
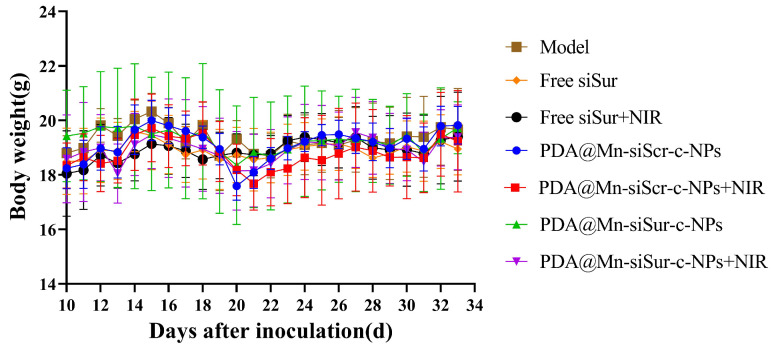
Body weight changes in mice of each group during the drug administration period (*n* = 5).

The changes in the weight of mice during the medication cycle were also recorded. As illustrated in Figure 8, the trend in weight change was consistent among all groups of mice, indicating that the formulations exerted only a minimal impact on the weight of the animals.

### 3.11. Western Blotting

**Figure 9:**
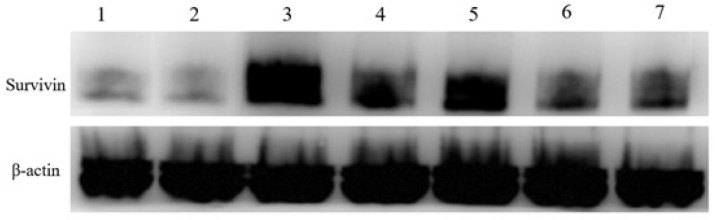
Western blotting results for tumor tissue of mice in each group (1: PDA@Mn-siSur-c-NPs+NIR, 2: PDA@Mn-siSur-c-NPs, 3: PDA@Mn-siScr-c-NPs+NIR, 4: PDA@Mn-siScr-c-NP, 5: Free siSur+NIR, 6: Free siSur, 7: Model).

Silencing survivin using siRNA is a primary means of treating tumors. Here, Western blotting was employed to examine the silencing effect of each formulation on survivin protein expression, with β-actin serving as a reference (Figure 9). The results showed that PDA@Mn-siSur-c-NPs had the best survivin silencing efficacy. Survivin protein expression increases following exposure to light, and silencing with PDA@Mn-siSur-c-NPs helps to counteract such a light exposure-induced increase in survivin protein expression, which is beneficial for promoting the apoptosis of cancer cells [58].

Western blotting experiments demonstrated that the nanoparticle-encapsulated siRNA successfully silenced survivin protein expression within tumors, indicating that the modified siRNA was protected from degradation and was successfully delivered into the cells. Our analysis of the antitumor effects of the different formulations on tumor-bearing mice indicated that there were significant differences in tumor suppression effects between the irradiated and non-irradiated groups; the siSur and siScr (scrambled siRNA) groups, and the formulation and free siSur groups. The best tumor suppression effect was observed in the PDA@Mn-siSur-c-NPs under irradiation group.

Notably, photothermal mediated by the PDA core and survivin silencing via siSur exhibit a synergistic antitumor relationship that amplifies therapeutic efficacy beyond individual modalities. PTT directly induces tumor cell necrosis through NIR laser-triggered hyperthermia [59], while concurrently triggering adaptive anti-apoptotic responses—including upregulation of survivin, which helps residual tumor cells evade cell death and limits treatment durability. By contrast, siSur specifically silences survivin expression, not only directly initiating mitochondrial-dependent apoptosis and cell cycle arrest but also diminishing cellular thermal tolerance by downregulating the expression of heat shock protein 70, which in turn markedly boosts the sensitivity of cancer cells to thermal damage [60,61].

### 3.12. Safety Evaluation

Manganese is an essential human trace element that aids metabolism and neurodevelopment. However, excessive exposure or poor elimination causes toxicity, linked to liver function. Impaired liver function blocks manganese’s biliary excretion, leading to accumulation that harms the central nervous system, causing Parkinsonism-like and neuropsychiatric symptoms. Most large studies confirm long-term occupational exposure raises this risk, and acute toxicity harms the liver and kidneys [62,63,64]. As for the measurement of biochemical indicators, compared to the model group, there were no significant differences in AST, ALT, BUN, or CRE activity among the mice in each group (Table 4). The AST in Free siSur+NIR and PDA@Mn-siSur-c-NPs groups were slightly higher, while the parameters were in the normal range [65], indicating that the formulations exhibited no significant hepato- or nephrotoxicity. Histological examination revealed (Figures S3–S8) that the myocardial fiber cells in the heart tissue of the mice in all the treatment groups were arranged similarly to those in the model group, with no evidence of heart damage. In the model group, the liver, spleen, and lung tissues of the mice had tightly packed cells without obvious pathological features. The mice in the treatment groups showed good integrity of liver, spleen, and lung cells, indicating that the formulations had low toxicity against these tissues. The tumor cells in mice of the model group were closely packed, regular in shape, and had small intercellular spaces. In contrast, tumor cells in the medication groups showed irregular morphology, indicative of cell damage, and, in the PDA@Mn-siScr-c-NPs+NIR, PDA@Mn-siSur-c-NPs+NIR, and Free siSur+NIR groups, the tumor cells were loosely arranged with increased intercellular spacing.

## 4. Conclusions

Established cancer therapies are constrained by single-modality insufficiency. However, given the complexity of tumor cells and the tumor microenvironment—characterized by spatiotemporally dynamic crosstalk, aberrant vascularization, immunosuppressive niches, and adaptive resistance mechanisms—a single therapeutic modality falls far short of addressing the multifaceted therapeutic challenges. Consequently, the rational design of multifunctional nano-drug delivery systems, which achieve combinatorial intervention against malignant tumors, has long remained a focus in cancer research.

In the present study, we successfully engineered a targeted therapeutic nanoformulation with a PDA@MnO_2_ as the core. To further optimize its biological performance, the core was sequentially functionalized: first with PAMAM to encapsulate siSur, enhance siRNA stability; then with cRGD peptide to achieve active targeting to α_v_β_3_ integrin-overexpressing tumor cells. Then, the final nanoparticle system, PDA@Mn-siSur-c-NPs, was yielded.

PDA@Mn-siSur-c-NPs exhibited excellent siRNA protection, low toxicity, and high photo-thermal conversion efficiency. The antitumor efficacy of this formulation upon NIR laser irradiation was carefully evaluated both in vitro and in vivo. The results showed that the combined treatment could achieve significant tumor regression in tumor-bearing mice. These findings indicate that, PDA@Mn-siSur-c-NPs, this biocompatible nanoformulation, offers a promising option for superficial and locally advanced tumors clinically, representing a targeted, synergistic approach that transcends the limitations of conventional therapies.

While these findings underscore the promise of our design, we acknowledge inherent considerations aligned with challenges: light penetration depth currently limits applicability to deep-seated or widely metastatic tumors, and long-term safety assessments remain to be explored—consistent with ongoing efforts in nanosystem clinical translation. These aspects not only reflect avenues for future optimization but also reinforce the value of our current work for advancing synergistic therapy with thermal-based therapies.

## Figures and Tables

**Table 1:** The hydrodynamic diameters, polydispersity indexes (PDIs), and zeta potentials of each formulation (means ± standard deviation).

Sample	Hydrodynamic Diameters (nm)	PDI	Zeta Potential (mV)
MnO_2_	35.73 ± 1.00	0.21 ± 0.04	0.07 ± 0.03
PDA@Mn	93.80 ± 4.85	0.16 ± 0.14	−13.80 ± 0.42
PDA@Mn-siSur-c-NPs	125.10 ± 2.07	0.14 ± 0.01	10.51 ± 1.21

**Table 2:** Cellular uptake of Mn in 4T1 cells.

Formulation	Mn Concentration After 5 min (μg/mL)	Mn Concentration After 15 min (μg/mL)
PDA@Mn-siSur-NPs	0.21 ± 0.09	0.27 ± 0.06
PDA@Mn-siSur-c-NPs	0.25 ± 0.03	0.34 ± 0.13

**Table 3:** Pharmacokinetic parameters of different formulations in rats (*n* = 3).

Group	AUC_0→t_ (ng/mL·h^−1^)	MRT (h)	CL (μg)/(ng/mL)/h	V_d_ (μg)/(ng/mL)
PDA@Mn-siSur-NPs	131.786 ± 12.686	1.646 ± 0.090	0.345 ± 0.038	1.513 ± 0.117
PDA@Mn-siSur-c-NPs	362.297 ± 33.587	5.006 ± 1.519	0.108 ± 0.043	1.227 ± 0.147

Abbreviation: AUC_0_**_→_**_t_, area under the concentration−time curve from zero to the final time point; MRT, mean residence time; CL, clearance; V_d_, volume of distribution. Data were shown as mean ± standard deviation.

**Table 4:** Biochemical indicators of mice (*n* = 3).

Groups	ALT (U/L)	AST (U/L)	CRE (μmol/L)	BUN (mmol/L)
Model	34.00 ± 8.19	252.00 ± 15.10	6.87 ± 15	10.83 ± 0.31
Free siSur	31.00 ± 11.36	240.33 ± 32.96	5.40 ± 1.44	7.33 ± 2.56
Free siSur+NIR	39.00 ± 6.93	331.50 ± 17.68	7.03 ± 1.89	8.63 ± 0.29
PDA@Mn-siScr-c-NPs	24.67 ± 12.58	288.67 ± 54.86	6.07 ± 1.07	10.10 ± 0.26
PDA@Mn-siScr-c-NPs+NIR	40.67 ± 13.65	261.67 ± 78.36	5.77 ± 0.6	8.60 ± 0.56
PDA@Mn-siSur-c-NPs	28.33 ± 18.01	305.5 ± 45.5	5.83 ± 1.45	9.73 ± 0.93
PDA@Mn-siSur-c-NPs+NIR	31.67 ± 11.24	266.00 ± 33.15	6.63 ± 0.57	9.63 ± 0.06

Abbreviations: ALT, alanine aminotransferase; AST, aspartate aminotransferase; CRE, creatinine; BUN, blood urea nitrogen.

## Data Availability

The original contributions presented in this study are included in the article/Supplementary Material. Further inquiries can be directed to the corresponding author(s).

## Supplementary Materials

The following supporting information can be downloaded at: https://www.mdpi.com/article/10.3390/pharmaceutics18010010/s1, Figure S1. Representative particle size and zeta potential distribution of formulations (MnO_2_, PDA@Mn, and PDA@Mn-siSur-c-NPs); Figure S2. High-resolution Mn2p XPS spectra of MnO_2_ and PDA@Mn; Figure S3. Hematoxylin and eosin (H&E) staining of heart tissues from mice in all the groups (A: Model group, B: Free siSur, C: Free siSur+NIR, D: PDA@Mn-siScr-c-NPs, E: PDA@Mn-siScr-c-NPs+NIR, F: PDA@Mn-siSur-c-NPs, G: PDA@Mn-siSur-c-NPs+NIR); Figure S4. Hematoxylin and eosin (H&E) staining of liver tissues from mice in all the groups (A: Model group, B: Free siSur, C: Free siSur+NIR, D: PDA@Mn-siScr-c-NPs, E: PDA@Mn-siScr-c-NPs+NIR, F: PDA@Mn-siSur-c-NPs, G: PDA@Mn-siSur-c-NPs+NIR); Figure S5. Hematoxylin and eosin (H&E) staining of spleen tissues from mice in all the groups (A: Model group, B: Free siSur, C: Free siSur+NIR, D: PDA@Mn-siScr-c-NPs, E: PDA@Mn-siScr-c-NPs+NIR, F: PDA@Mn-siSur-c-NPs, G: PDA@Mn-siSur-c-NPs+NIR); Figure S6. Hematoxylin and eosin (H&E) staining of lung tissues from mice in all the groups (A: Model group, B: Free siSur, C: Free siSur+NIR, D: PDA@Mn-siScr-c-NPs, E: PDA@Mn-siScr-c-NPs+NIR, F: PDA@Mn-siSur-c-NPs, G: PDA@Mn-siSur-c-NPs+NIR); Figure S7. Hematoxylin and eosin (H&E) staining of kidney tissues from mice in all the groups (A: Model group, B: Free siSur, C: Free siSur+NIR, D: PDA@Mn-siScr-c-NPs, E: PDA@Mn-siScr-c-NPs+NIR, F: PDA@Mn-siSur-c-NPs, G: PDA@Mn-siSur-c-NPs+NIR); Figure S8. Hematoxylin and eosin (H&E) staining of tumor tissues from mice in all the groups (A: Model group, B: Free siSur, C: Free siSur+NIR, D: PDA@Mn-siScr-c-NPs, E: PDA@Mn-siScr-c-NPs+NIR, F: PDA@Mn-siSur-c-NPs, G: PDA@Mn-siSur-c-NPs+NIR).
