# Supplementary material for: Construction and Characterization of PDA@MnO2-Cored Multifunctional Targeting Nanoparticles Loaded with Survivin siRNA for Breast Tumor Therapy
Source: Pharmaceutics. 2025 Dec 21;18(1):10. doi: 10.3390/pharmaceutics18010010 (PMC12844724; doi:10.3390/pharmaceutics18010010)
Supplement: Supplementary file 1 [file pharmaceutics-18-00010-s001.zip › pharmaceutics-3958327-supplementary.pdf]

Supplementary materials

Construction and Characterization of PDA@MnO_2-Cored Multifunctional Targeting Nanoparticles Loaded with Survivin siRNA for Breast Tumor Therapy

Methods

X-ray photoelectron spectroscopy analysis

X-ray Photoelectron Spectroscopy (XPS) characterization was performed using a Thermo Scientific K-Alpha spectrometer (ThermoFisher, MA, US) equipped with an Al Kα X-ray source, operating with a 400 μm beam spot, an analysis chamber vacuum of >5.0×10^{-7} mbar, a 12 kV voltage, and a 6 mA filament current; survey scans were acquired with a 150 eV pass energy and 1 eV step, while narrow scans used a 50 eV pass energy, 0.1 eV step. The MnO_2 and PDA@Mn particles were prepared, collected and dried. Then, these solid powder samples were pressed into pellets, mounted on holders for testing.

Results

XPS analysis focused on the Mn2p high-resolution spectra to evaluate the valence state of Mn in the samples (Fig. S2). The Mn2p peak of MnO_2 was located at 641.09 eV (with a full width at half maximum, FWHM, of 3.35 eV), while that of PDA@Mn appeared at 640.79 eV (FWHM = 3.23 eV). Specifically, the Mn2p peak positions of both samples fall within the characteristic binding energy interval of Mn^{4+} in MnO_2, directly confirming that the valence state of Mn has not changed by coating with PDA.

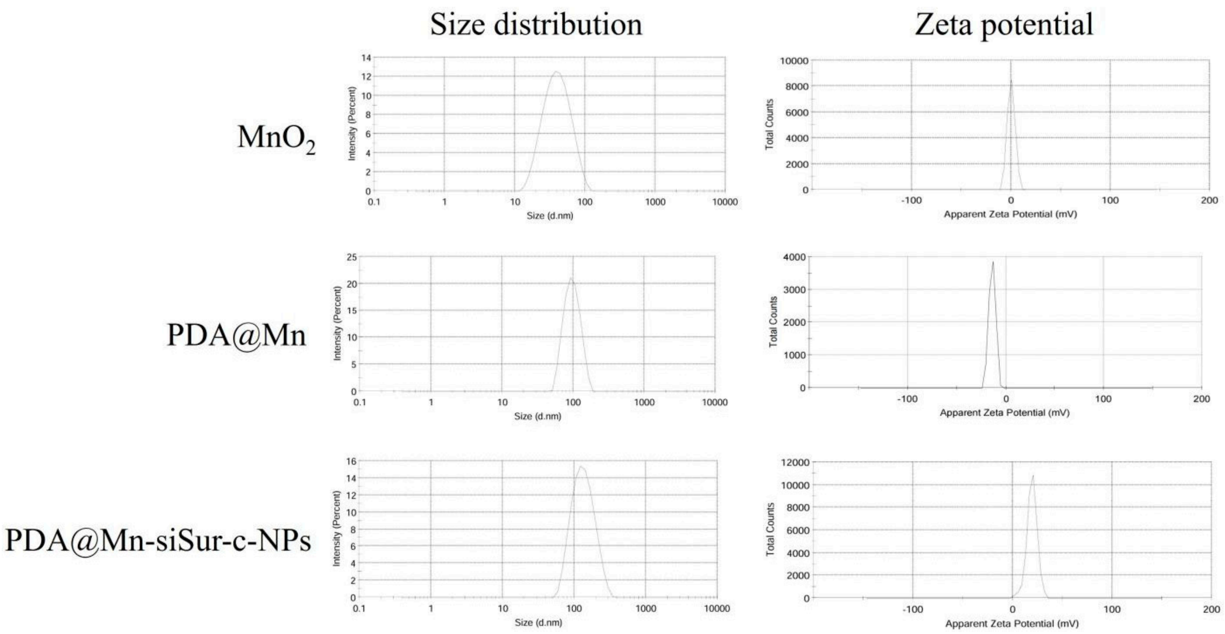

Fig. S1. Representative particle size and zeta potential distribution of formulations (MnO_2, PDA@Mn, and PDA@Mn-siSur-c-NPs).

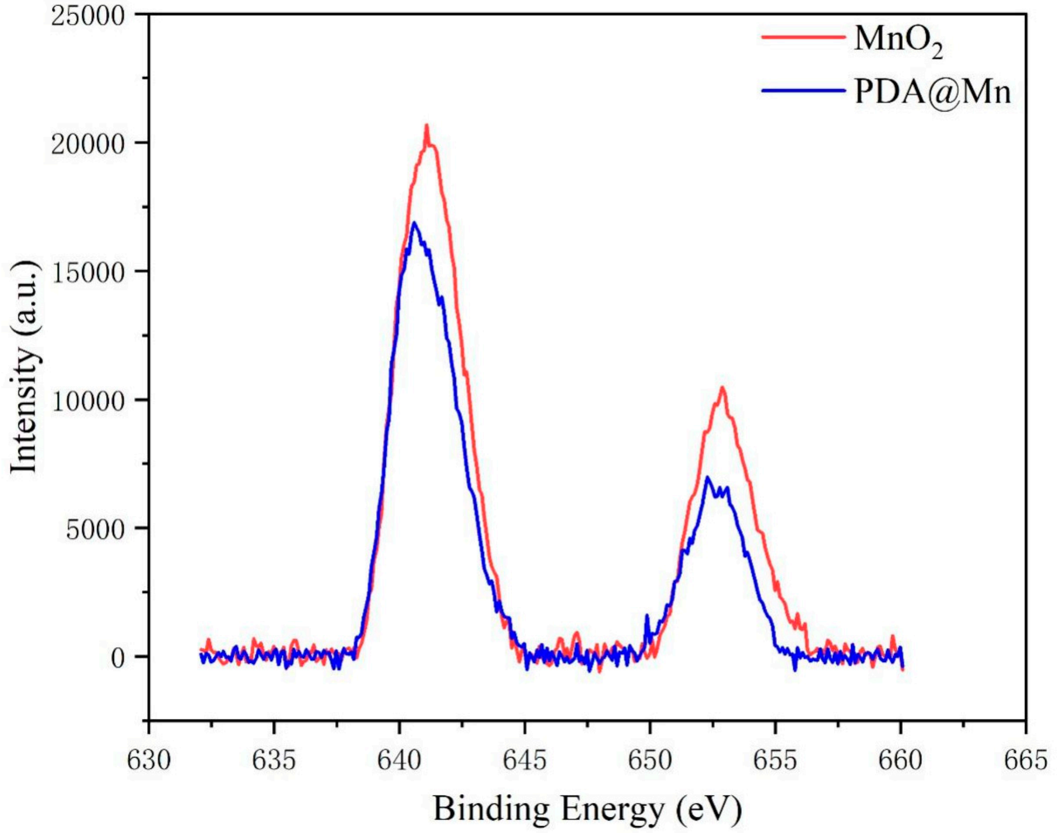

Fig. S2. High-resolution Mn2p XPS spectra of MnO_2 and PDA@Mn.

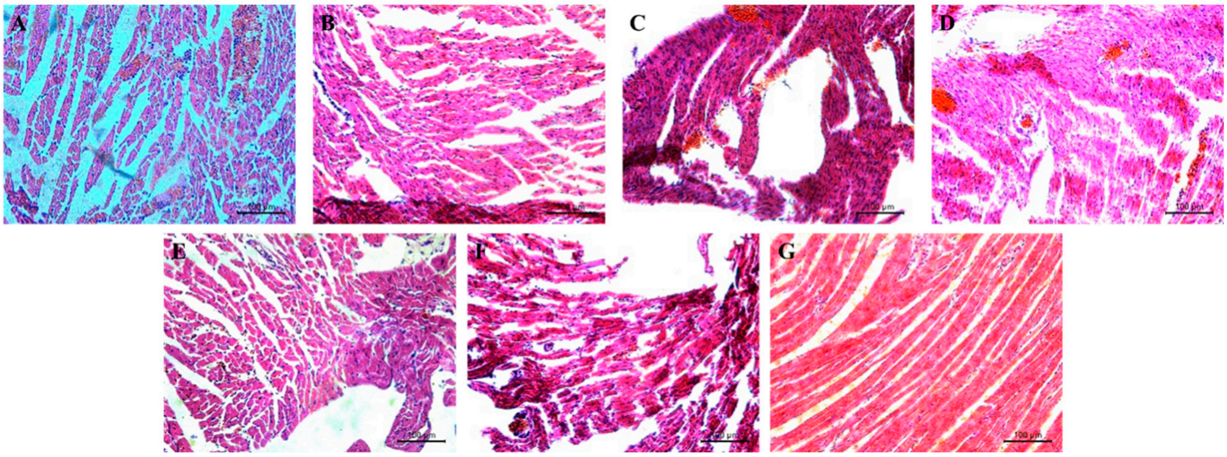

Fig. S3. Hematoxylin and eosin (H&E) staining of heart tissues from mice in all the groups (A: Model group, B: Free siSur, C: Free siSur+NIR, D: PDA@Mn-siScr-c-NPs, E: PDA@Mn-siScr-c-NPs+NIR, F: PDA@Mn-siSur-c-NPs, G: PDA@Mn-siSur-c-NPs+NIR).

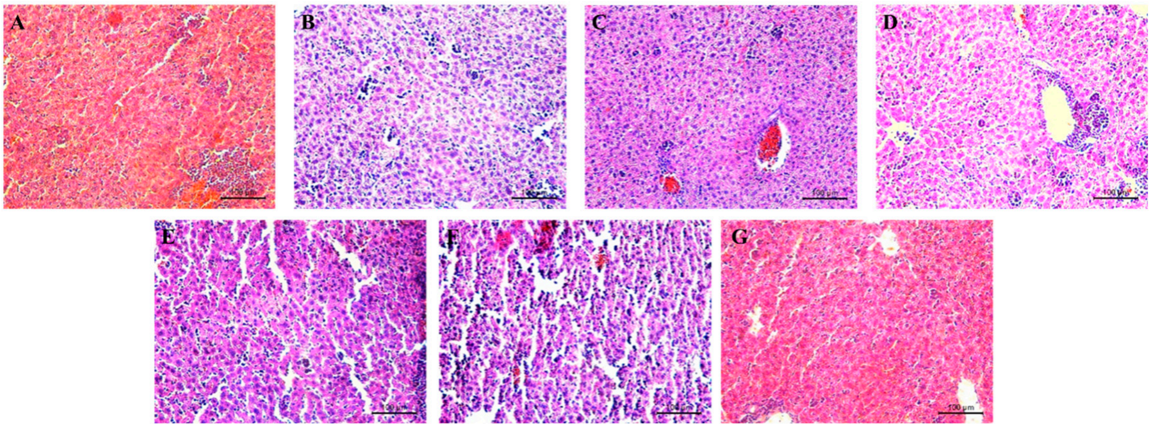

Fig. S4. Hematoxylin and eosin (H&E) staining of liver tissues from mice in all the groups (A: Model group, B: Free siSur, C: Free siSur+NIR, D: PDA@Mn-siScr-c-NPs, E: PDA@Mn-siScr-c-NPs+NIR, F: PDA@Mn-siSur-c-NPs, G: PDA@Mn-siSur-c-NPs+NIR).

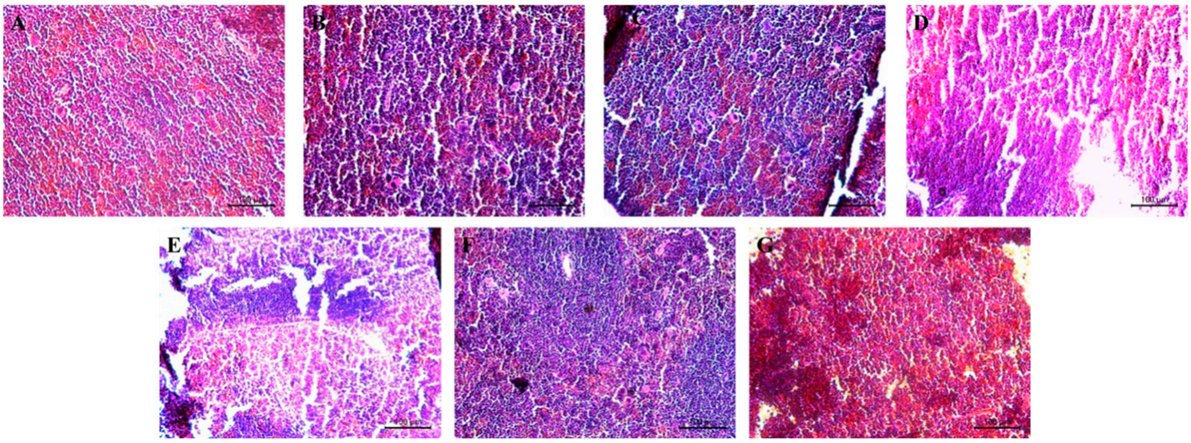

Fig. S5. Hematoxylin and eosin (H&E) staining of spleen tissues from mice in all the groups (A: Model group, B: Free siSur, C: Free siSur+NIR, D: PDA@Mn-siScr-c-NPs, E: PDA@Mn-siScr-c-NPs+NIR, F: PDA@Mn-siSur-c-NPs, G: PDA@Mn-siSur-c-NPs+NIR).

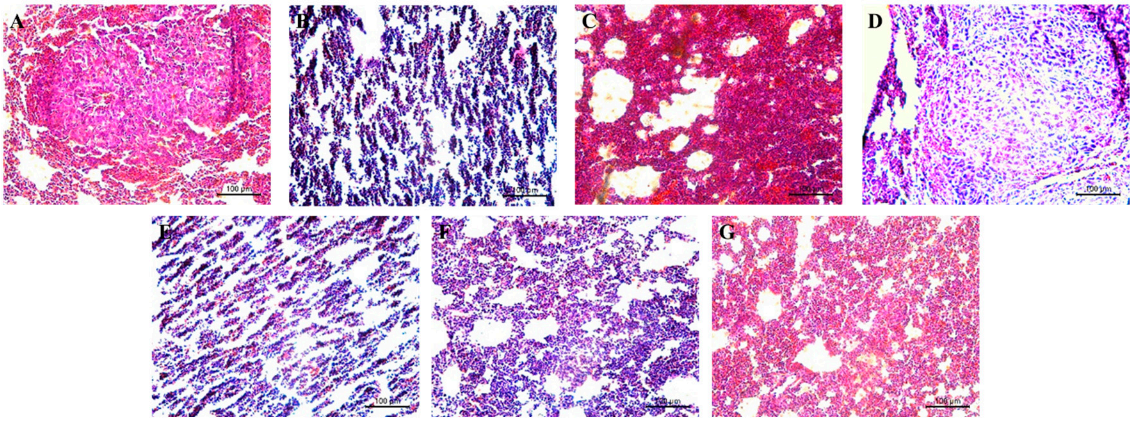

Fig. S6. Hematoxylin and eosin (H&E) staining of lung tissues from mice in all the groups (A: Model group, B: Free siSur, C: Free siSur+NIR, D: PDA@Mn-siScr-c-NPs, E: PDA@Mn-siScr-c-NPs+NIR, F: PDA@Mn-siSur-c-NPs, G: PDA@Mn-siSur-c-NPs+NIR).

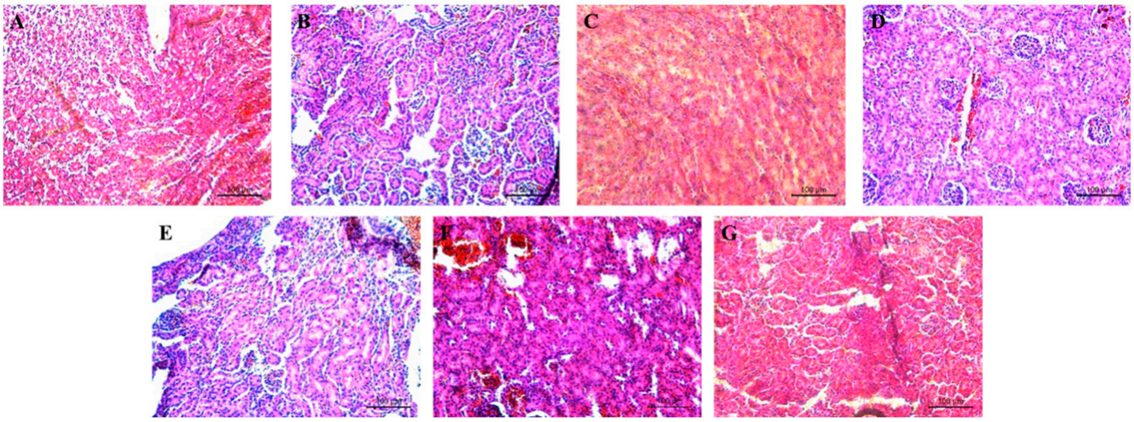

Fig. S7. Hematoxylin and eosin (H&E) staining of kidney tissues from mice in all the groups (A: Model group, B: Free siSur, C: Free siSur+NIR, D: PDA@Mn-siScr-c-NPs, E: PDA@Mn-siScr-c-NPs+NIR, F: PDA@Mn-siSur-c-NPs, G: PDA@Mn-siSur-c-NPs+NIR).

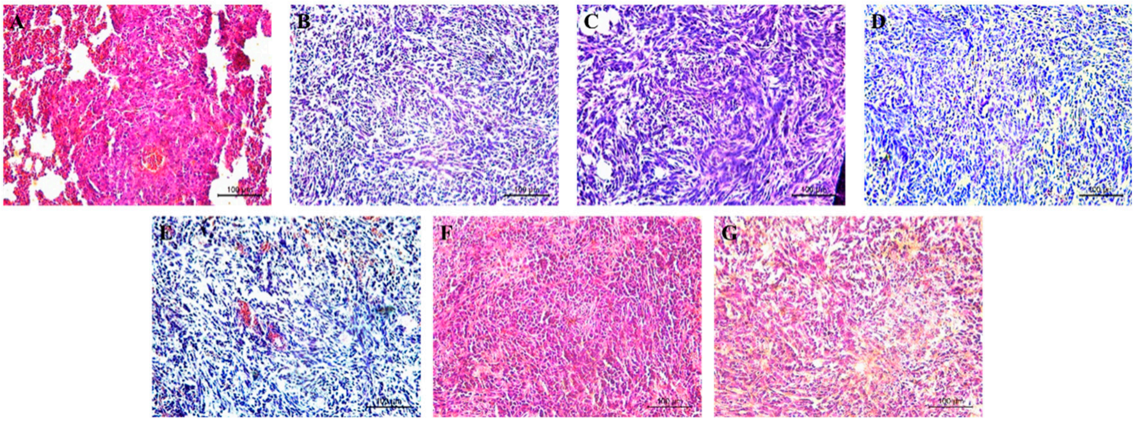

Fig. S8. Hematoxylin and eosin (H&E) staining of tumor tissues from mice in all the groups (A: Model group, B: Free siSur, C: Free siSur+NIR, D: PDA@Mn-siScr-c-NPs, E: PDA@Mn-siScr-c-NPs+NIR, F: PDA@Mn-siSur-c-NPs, G: PDA@Mn-siSur-c-NPs+NIR).
